# Anxious Solitude, Reciprocated Friendships with Peers, and Maternal Overcontrol from Third through Seventh Grade: A Transactional Model

**DOI:** 10.3390/children8050379

**Published:** 2021-05-11

**Authors:** Heidi Gazelle, Ming Cui

**Affiliations:** Department of Human Development and Family Science, College of Health and Human Sciences, Florida State University, Tallahassee, FL 32306-1491, USA; mcui@fsu.edu

**Keywords:** social withdrawal, social anxiety, shyness, friends, peers, maternal overcontrol, transaction, middle school transition, longitudinal study, child development

## Abstract

Guided by a *Transactional Model* of anxious solitude development, we tested friend and maternal influences on continuity and change in youth anxious solitude from 3rd through 7th grade, as well as the influence of youth anxious solitude on decreased friendship participation and increased maternal overcontrol over time. Participants were 230 American youth (57% girls) selected for longitudinal study from a public-school screening sample (*n* = 688). Peers reported on anxious solitude, both peers and youth reported on reciprocated friendship, and youth reported on their mother’s overcontrol annually. Stability and incremental change in youth, friend, and maternal factors were tested in an *autoregressive cross-lagged panel analytic model*. Having few mutual friendships predicted incremental increase in youth anxious solitude in mid-elementary school, then youth anxious solitude predicted the loss of friendships after the middle school transition. Additionally, youth anxious solitude in third grade evoked increased maternal overcontrol in fourth grade, but the reverse direction of effect was not supported. Youth’s participation in few friendships also evoked mothers’ overcontrol, which exacerbated their child’s loss of friendships in elementary school. Taken together, having few mutual friends contributed to youth anxious solitude and maternal overcontrol, and subsequently these factors further exacerbated youth’s loss of friendships.

## 1. Introduction

Youth’s closest relationships are with their friends and parents in the middle childhood to early adolescent period [1]. Consequently, youth’s participation in friendships and the nature of parenting they experience in this period may influence continuity and change in their anxious solitude [2]. Anxious solitary youth demonstrate shy, socially anxious behavior, and low rates of interaction with familiar peers (e.g., classmates) [3]. In this investigation we evaluate the impact of youth’s friendships (or the lack thereof) and maternal overcontrol on the development of their anxious solitude from middle childhood through early adolescence [2]. Likewise, we evaluate whether youth’s anxious solitude predicts decreased friendship participation and increased maternal overcontrol over time. We will also explore potential relations between youth’s friendship participation and the maternal overcontrol they experience over time.

This study was guided by a *Transactional Model* of youth anxious solitude development in which interpersonal stress (losing friendships, and maternal overcontrol) contributes to growth in youth anxious solitude from middle childhood through early adolescence, and conversely growth in youth anxious solitude also exacerbates interpersonal stress over time. Our *Transactional Model* also posits that mothering is responsive not only to youth’s behavior, but also to youth’s social adjustment as manifested in friendships. We examine these relations over a five-year period from 3rd through 7th grade, which includes the transition to middle school in the fall of sixth grade.

### 1.1. Youth Anxious Solitude

*Anxious solitude* is an affective-behavioral profile in which youth frequently remain alone when among familiar peers (e.g., classmates) due to social anxiety [3]. Anxious solitary children are conceptualized as wanting to interact with their peers (normative social approach motivation) but being impeded from doing so by their social anxiety. This social anxiety is believed to be rooted in the youth’s concerns that s/he may not be well received by peers or interact competently. Consequently, when anxious solitary children are among familiar peers, relative to age mates they display elevated rates of solitary onlooking behavior (watching peers’ interactions without joining in), solitary unoccupied behavior (wandering aimlessly, and staring into space), and verbal reticence (saying little to peers). About 10 to 15% of children from community samples demonstrate elevated anxious solitude [3]. Anxious solitude is not a diagnosis, but about a third of anxious solitary children experience sufficient impairment to receive a diagnosis of social anxiety disorder [4].

Anxious solitude falls under the umbrella term “social withdrawal,” which describes elevated rates of solitary behavior relative to age mates. For anxious solitary youth, social withdrawal is driven by social anxiety, but social withdrawal can also be driven by social disinterest for unsociable youth [5] or by depression [6]. The current paper focuses on anxious solitude because it is the most common form of social withdrawal and has been linked with both low friendship participation [7,8] and maternal overcontrol [9].

### 1.2. Direction of Effect in the Relation between Youth Anxious Solitude and Interpersonal Stress over Time

To test the proposed *Transactional Model*, it is necessary to evaluate the direction of effect between each of the following three constructs over time: youth anxious solitude, reciprocated friendship, and maternal overcontrol. Therefore, testing this model requires evaluating *Chronic Stress effects* [10], in which losing (and not replacing) friends and/or increased maternal overcontrol over time may contribute to incremental increase in youth anxious solitude over time, as well as *youth Stress Generation effects* [11], in which increased youth anxious solitude may contribute to losing (and not replacing) friends and/or evoking increased maternal control over time (see Table 1).

#### 1.2.1. Direction of Effect in the Relation between Youth Anxious Solitude and Friendship

*Does having few mutual friends contribute to increasing youth anxious solitude in middle childhood and early adolescence (peer-driven Chronic Stress effects)?* Friendships are dyadic peer relationships characterized by mutual liking and companionship (spending time together), and typically involve emotional intimacy and loyalty in later middle childhood and early adolescence. Additionally, friends provide youth with validation and instrumental help [12]. Therefore, youth who have few friends—as is the case for many anxious solitary youth [7,8]—may not have their fundamental psychological and practical needs met [13]. Inability to satisfy needs for affiliation and belonging may leave youth feeling vulnerable among peers and contribute to increased anxious solitude over time. For example, a youth who has few friends in comparison to peers may feel unsure of how peers will respond to his or her overtures and lack confidence in initiating interactions with peers and joining into peer activities.

Furthermore, youth’s interactions with their friends are more complex and rewarding than those with other peers [14]. Therefore, youth who have few friends may become increasingly out-of-step with peers in developing age-appropriate social skills and social knowledge over time because they have little opportunity to practice these skills and gain knowledge about peer culture in the context of friendship. Such social immaturity may foster anxious solitude by contributing to painful social experiences (e.g., social gaffs which result in teasing) which exacerbate youth’s social evaluative concerns.

In support of *peer-driven Chronic Stress effects*, evidence from three previous investigations suggests that friendlessness [15,16] and little positive interaction with friends [17] predict *increasing* anxious solitude in middle childhood and early adolescence. However, one previous study did *not* find such a relation [18] and another of these studies found that friendlessness no longer significantly predicted increasing anxious solitude trajectories in elementary school after controlling for ability to defend oneself, externalizing behavior, and gender moderation [15]. Furthermore, it is unclear whether relatively low friendship participation rates (having relatively few friends or losing some friends over time, and not only complete friendlessness) would also predict increased anxious solitude over time. Importantly, investigations that have found that friendlessness predicts increased anxious solitude over time typically have not examined the reverse direction of effect—that is, they have not examined whether anxious solitude leads to friendlessness or losing friends (without replacing them) over time.

*Does youth’s anxious solitude predict decreasing numbers of mutual friendships over the course of middle childhood and early adolescence (youth Stress Generation effects)?* Anxious solitary behavior may interfere with youth’s ability to maintain friendships because the friends of anxious solitary youth perceive their friendships to be less close and helpful on average than does the anxious solitary youth, and observations reveal that anxious solitary youth—in comparison to their peers—are less communicative with their friend [19] (see also [20]). Such limitations may negatively impact friendship satisfaction, the amount of time friends spend together, and the longevity of friendships. Thus, anxious solitude may contribute to losing friends (without replacing them) over the course of middle childhood and early adolescence.

In regard to evidence for *youth Stress Generation effects*, little is known about whether anxious solitude contributes to declining friendship participation over time. Two studies found that characteristics that partially comprise anxious solitude (shyness, timidity) did *not* predict friendship loss during the course of an academic year [18] or summer camp [21], although anxious solitude is related to low friendship participation [7,8,21]. However, it is possible that more robust measures of anxious solitude might yield different results. Moreover, anxious solitude could lead to friendship loss over longer durations, such as multiple school years.

#### 1.2.2. Direction of Effect in the Relation between Youth Anxious Solitude and Maternal Overcontrol

*Does maternal overcontrol contribute to increasing youth anxious solitude in middle childhood and early adolescence (maternally-driven Chronic Stress effects)?* Maternal overcontrol describes parenting practices in which the mother does *not* allow her child to make choices about how to do things nor convey to her child that she cares about how he or she feels and thinks about things. Thus, mothers who are overcontrolling allow their child little autonomy and convey little regard for their child’s perspectives [22]. Maternal overcontrol is believed to contribute to anxious solitude because it suggests to the youth that s/he is not socially competent and may restrict her/his exposure to social interaction [9]. As a consequence, the youth may lack confidence and be hesitant to share their ideas and interact with others. Therefore, they are not likely to have interactions and get feedback that would support growth in their maturity and awareness of others’ perspectives over time. As a result, they may appear increasingly immature relative to their peers over time.

In regard to evidence for *maternally-driven Chronic Stress effects*, maternal overcontrol has been shown to predict *greater stability* in youth anxiety over two-and-a-half years during the transition to early adolescence [23], and *greater increases* in anxious solitude among youth with high-increasing anxious solitude trajectories in 5th to 8th grade [24]. However, maternal overcontrol did *not* contribute to incremental increases in youth anxious solitude from year-to-year during late middle childhood and early adolescence in a recent study that directly tested these effects [9]. To evaluate a transactional model, it is also important to consider the reverse direction of effect.

*Do increases in youth’s anxious solitude predict increasing maternal overcontrol over the course of middle childhood and early adolescence (youth Stress Generation effects)?* Youth anxious solitude may engender concern in mothers, and some mothers may respond with overcontrol in an attempt to prevent their child from feeling anxious. In support of such youth *Stress Generation effects*, a recent study found that anxious solitude predicted significant increases in maternal overcontrol from year-to-year during elementary school [9,25,26,27,28]. Other investigations also suggest evocative effects of child anxiety on parenting [25,26,27,28].

Taken together, we expected evidence for the relation between youth anxious solitude and maternal overcontrol in the middle childhood to early adolescent period to support *youth Stress Generation effects* but not *maternally-driven Chronic Stress effects*. That is, in the middle childhood to early adolescent period, we expected anxious solitude to evoke maternal overcontrol, rather than vice versa.

#### 1.2.3. Direction of Effect in the Relation between Youth’s Friendships and Maternal Overcontrol

*Does mothers’ awareness that their child has few or declining numbers of mutual friends contribute to increasing maternal overcontrol in middle childhood and early adolescence (peer-driven relational Stress Generation*, see Table 1*)?* Mothers recognize the importance of friendships to their children’s healthy psychosocial development. When youth have few or declining numbers of friends, mothers may be concerned and express such concern through overcontrolling parenting. Such friend effects have not been tested, as few investigations consider the possibility that mothers may be influenced by their child’s peer relationships. However, one recent study found that the peer exclusion of one’s child indirectly evoked increased maternal overcontrol over time via increased youth anxious solitude in late middle childhood [9].

*Does increasing maternal overcontrol contribute to decreases in youth’s mutual friendships over the course of middle childhood and early adolescence (maternally-driven relational Stress Generation)?* Maternal overcontrol may contribute to decreasing friendships over time because it could directly limit youth’s opportunities to interact with friends (e.g., they are not allowed to have friends spend the night) or indirectly limit youth’s friendship competency through undermining their social confidence. Both of these potential directions of effect will be tested in the proposed *Transactional Model*.

### 1.3. The Middle School Transition and Gender Differences

The middle childhood to early adolescent time period covered by the current investigation includes the middle school transition in the fall of sixth grade. The middle school transition is typically considered a stressful time for young adolescents, as it requires adapting to a larger school environment in which students change classes and consequently may have fewer close relationships with teachers and must find their place among a larger group of peers [29]. Girls in particular have been thought to encounter stress after the transition to middle school because it more closely corresponds with the pubertal transition for many girls, leading to the need to adapt to multiple challenges at once. Nonetheless, evidence suggests that anxious solitary youth experience a drop in peer exclusion after the middle school transition due to the collective renegotiation of peer groups [30]. However, it is unclear how anxious solitary youth may fare with their friendships after the middle school transition, and whether anxious solitary girls in particular may encounter challenges with maintaining friendships after the middle school transition [31].

### 1.4. Effects of Friendship While Controlling for Peer Exclusion

In this investigation, we also evaluated the impact of youth’s friendships (or the lack thereof) on the development of their anxious solitude while accounting for the effects of peer exclusion [2], because evidence indicates that peer exclusion contributes to increase in anxious solitude over time [9]. Peer exclusion is a group-level phenomenon in which children are left out of their classmates’ interactions [3]. We have proposed that peer exclusion contributes to anxious solitude via a diathesis (vulnerability)-stress process in which peer exclusion confirms anxious solitary children’s social fears [3]. Nonetheless, evidence suggests that dyadic friendships among peers and group-level peer relations such as peer exclusion each make unique contributions to youth’s psychosocial adjustment [32]. Therefore, it is important to examine whether having few or declining numbers of friends contributes to increases in anxious solitude above and beyond the effects of peer exclusion.

### 1.5. Expected Results

To test the proposed *Transactional Model*, we will evaluate *Chronic Stress effects*, in which losing (and not replacing) friends and/or increased maternal overcontrol over time may contribute to incremental increase in youth anxious solitude over time, as well as *youth Stress Generation effects* [11], in which increased youth anxious solitude may contribute to losing (and not replacing) friends and/or evoking increased maternal control over time. Based on our previous research and the literature reviewed above, we expected evidence consistent with both *peer-driven Chronic Stress* (with friends) and *youth Stress Generation effects* (with both friends and mothers), in support of an overarching *Transactional Model* of anxious solitude development [9]. Relations between youth’s friendship participation and maternal overcontrol will also be examined.

## 2. Method

Youth were prospectively followed from 3rd through 7th grade.

### 2.1. Participants and Overview of Participant Selection Procedures

Participants were 230 selected youth with active informed parental consent who attended seven public elementary schools in 3rd grade in a suburban to rural region of the Southeastern United States. Youth of both sexes were similarly represented (57% girls, *n* = 130, χ^2^ = 4.45, *ns*). These participants were selected for multi-method longitudinal study from a larger peer-nomination screening sample (*n* = 688 in 3rd grade). Approximately half of this sample of 230 youth were selected for elevated peer-reported anxious solitude (scored *at or above* +1 *SD* relative to the larger screening sample) in 3rd grade (or subsequent time points). A +1 *SD* cutoff is typical in extant literature [20].

The other half of the sample of 230 was selected for scoring anywhere *below* the +1 *SD* cutoff on peer-reported anxious solitude in 3rd grade (or subsequent timepoints) and as demographic matches for youth selected for anxious solitude (in regard to gender, age, ethnicity, socioeconomic status (SES), and initial classroom). Thus, the other half of the sample was comprised of youth who scored *at all levels of anxious solitude* below the “high anxious solitude” cutoff. Therefore, the other half of the sample were youth who represent typical variation in anxious solitude found in the community, including mildly elevated, average, and low levels (but *not* as uniformly *low* in anxious solitude).

This selection strategy preserved the full distribution of anxious solitude while ensuring substantial representation of youth high in anxious solitude, and eliminated potential demographic confounds between youth above and below the threshold for anxious solitude. Preserving the full distribution of anxious solitude facilitates analyses in which anxious solitude is handled as a continuous variable.

Youth selected vs. non-selected for the longitudinal study did not significantly differ at the 3rd grade outset of the study in age (selected *M* = 8.68 years, *SD* = 0.50; non-selected *M* = 8.65 years, *SD* = 0.48; *t* = −0.73, *ns*) or SES (free or reduced lunch status: selected 30%, non-selected 30%; χ^2^ = 1.54, *ns*). However, girls were over-represented in the selected compared to the non-selected screening sample (57% in the selected sample vs. 49% in the non-selected sample, χ^2^ = 4.41, *p* < 0.05). This is consistent with the higher prevalence of anxious solitude in girls compared to boys in some other later childhood and adolescent samples [20]. Although the selected and non-selected screening samples were ethnically diverse and representative of the region (60% vs. 62% European American, 23% vs. 13% Latinx, 16% vs. 22% African American, and 1% vs. 2% Asian American, respectively), there were significant differences in the representation of two ethnic groups (χ^2^ = 13.51, *p* < 0.01). There were more Latinx (23% vs. 13%, *p* < 0.05) and fewer African Americans (16% vs. 22%, *p* < 0.05) in the selected sample relative to the non-selected screening sample due to the prevalence of anxious solitude in these groups.

### 2.2. The Longitudinal Design, Longitudinal Assessment Procedures, and Selection Procedures for Subsequent Addition of Selected Participants

Selected youth (*n* = 230) were prospectively followed for five years from 3rd through 7th grades. The middle school transition occurred in the fall of 6th grade. Peer nominations for anxious solitude, friendship, and peer exclusion were collected for the screening sample (and the selected sample which was a subset of the screening sample) in the fall and spring of 3rd grade (*n* = 688). Subsequently, from the 4th through 7th grades, peer nominations were gathered from selected participants and their classmates (or middle school grade mates in 6th and 7th grades) each fall and spring.

Because many of the non-selected screening sample participants continued to be the classmates or middle school grade mates of youth in the selected sample, they continued to participate in peer nominations at subsequent time points. If they later emerged as above threshold for peer-reported anxious solitude (or a demographic match for such a child who was themselves below threshold for anxious solitude) they were added to the selected sample. The majority of children were added in elementary school, with 163/230 present in 3rd grade, 34 added in 4th grade, 30 added in 5th grade, and the remaining 3 added in middle school. Such delayed additions to the selected sample nonetheless had peer-reported anxious solitude and friendship data from 3rd grade and subsequent time points which was included in analyses, but they did not provide self-reported maternal overcontrol data until they joined the selected sample.

Youth in the selected sample reported on maternal overcontrol each year in the fall of 3rd grade and in the spring of 4th through 7th grades. Therefore, peer report data from the fall of 3rd grade and the spring of 4th through 7th grades were analyzed in this report so that all data analyzed were collected concurrently at each annual assessment. These data collection points were separated approximately by one year (or longer for the gap from the fall of 3rd grade to the spring of 4th grade). Descriptive statistics were calculated with SPSS 27 [33]. See Table 2 for means, standard deviations, sample size and inter-correlations by grade and measure. The distribution of the data was assessed for Skewness and Kurtosis, and the results generally supported normality [34]. Stability *r*s are also listed under each measure description below.

Of the 230 selected participants, data for the assessments relevant to this report were available for 70% to 100% in 3rd grade, 79% to 86% in 4th grade, 90% to 98% in 5th grade; and after the middle school transition 78% to 84% in 6th grade, and 74% to 77% in 7th grade (see *n* by time point and assessment in Table 2). Missing data is most prevalent in 3rd grade because only 163 children had been selected into the longitudinal sample (and therefore provided self-reported maternal overcontrol data) at the first time point, with additional participants from the original screening sample added at subsequent time points as youth who had originally been below threshold for anxious solitude subsequently scored above threshold (or were selected as matched comparison youth). Little’s MCAR test indicated data were missing-completely-at-random over the five years.

### 2.3. Measures and Measure-Specific Procedures

Selected youth participated in peer nominations and youth reports at least yearly throughout the study.

#### 2.3.1. Peer-Report Nominations and Procedures

Nominations for anxious solitude, friendship, and peer exclusion were administered simultaneously to assenting youth in each classroom in the fall and spring of the 3rd through 7th grades. Nominations were read aloud to all participating youth in each classroom by trained research assistants and youth selected classmates’ (or middle school grade mates’) names on individual rosters. Nominations were unlimited and cross-sex nominations were allowed because they yield better psychometric properties than alternative approaches [35]. In elementary school, rosters listed the names of all youth with parental consent in the *class* (Mean *n* = 15–18, *n* range = 8–24). In middle school, because youth had different sets of classmates for different classes, rosters listed names of youth with parental consent in the *grade* (Mean *n* = 121–135, *n* range = 10–250). However, youth attended their core classes with grade mates who were on the same *“team”* (a way of organizing middle school students into groups within grades so that they primarily attend class and interact with a smaller set of peers—these teams are *not* sports related). Therefore, only nominations from middle school teammates (Mean *n* = 52–65, range = 3–89) were included in the calculation of middle school composites because middle school students spent most of the day with teammates, and therefore were likely to be more familiar with their behavior.

Our conservative aim was to achieve at least 70% participation in peer nominations of students per classroom to ensure that nominations were representative of classmates’ perspectives, although research indicates that most peer nominations are internally reliable with lower participation rates [36]. In elementary school our target participation rate was achieved or exceeded in 98% (45/46) of third grade classrooms (*M* = 80% participation by classroom), 91% (50/55) of fourth grade classrooms (*M* = 76% participation by classroom), and 83% (43/52) of fifth grade classrooms (*M* = 74% participation by class-room). In middle school a mean of 66% of grade mates by school participated in peer nominations in both sixth and seventh grades, which evidence indicates also ensures reliability [36].

Nominations were adapted from previous investigations [3]. Minor modifications in wording [in brackets] were made in middle school to ensure language was developmentally appropriate. Peer nomination composites were calculated as the mean of peer nominations received by each youth for each nomination item at each time point. For anxious solitude and peer exclusion, prior to computing peer nomination composite means, nominations received for each item were standardized by classroom (or middle school team) to control for variations in size of elementary school classrooms and middle school teams. For reciprocated friendship, the single-item (*not* mean) composite was calculated as the sum of reciprocated nominations for each youth which was *not* standardized by classroom, because the sum of youth’s raw number of friends is inherently meaningful.

**Anxious Solitude.** The anxious solitude composite was comprised of three peer nominations that are well-established indices of anxious solitude [3,4,5,7,8,9]: “children who …” (1) “…act really shy around other kids. They seem to be nervous or afraid to be around other kids and they don’t talk much. They often play alone at recess [At lunch they often sit alone or don’t have anyone to talk to]”; (2) “… watch what other kids are doing but don’t join in. At recess they watch other kids playing but they play by themselves [At lunch they watch other kids talking but don’t join into the conversation]”; and (3) “…are very quiet. They don’t have much to say to other kids” (*Ms* = 0.21–0.45, *SDs* = 1.03–1.50). This composite demonstrated adequate reliability at each time point (αs = 0.76–0.96) and stability between successive time points (*r*s = 0.41–77, *p*s *<* 0.01).

**Reciprocated Friendship.** Peers nominated an unlimited number of classmates who “…are your close friends.” No restraints were placed on the gender of friendship pairs. When youth chose each other as a close friend (regardless of rank), this was counted as a *reciprocated friend* (*M* number of reciprocated friends = 1.45–2.95, *SDs* = 1.24–2.38). In Table 2 the mean number of reciprocated friends is higher in the fifth grade and thereafter, when youth were also asked to rank their closest friends (up to three). We think youth nominated more friends when they were also asked to rank friends because this prompted them to spend more time thinking about who their friends were, and they therefore were more thorough in nominating their friends.

**Peer Exclusion.** The peer exclusion composite consisted of two peer nominations “children who…” (1) “get left out when other kids are talking or playing [hanging out] together. They don’t get invited to parties or chosen to be on teams or to be work partners,” (2) “ask if they can play [hang out] and other kids say ‘no’ and won’t let them” (*Ms* = 0.16–0.29, *SDs* = 1.06–1.46). The composite was reliable (αs = 0.78–0.95) and stable between successive time points (*r*s = 0.51–0.72, *p*s *<* 0.01). Peer exclusion and anxious solitude were positively correlated as anticipated (*r*s = 0.49–0.83 at concurrent time points, *p*s *<* 0.01). Nonetheless, in a multi-trait, multi-method matrix in which items assessing these constructs were rated by five informants, peer exclusion and anxious solitude loaded on separate factors, supporting their divergent validity [5].

#### 2.3.2. Youth-Report Measure and Procedures

Youth-report measures were administered by trained research assistants who read questionnaires aloud to groups of five or fewer assenting youth in a quiet area of their school while youth marked their responses on individual questionnaires.

**Maternal Overcontrol.** Youth reported on their mother’s overcontrol on the autonomy-granting versus psychological control factor subscale on an abbreviated version of the revised *Child’s Report of Parental Behavior Inventory* (CRPBI [22]). The maternal overcontrol subscale consisted of 7 of the 16 items on the autonomy-granting versus psychological control subscale to permit administration in the time available. This subscale consisted of the following 7 items: “Gives me the choice of what to do whenever possible,” “Likes me to choose my own way of doing things whenever possible,” “Lets me help to decide how to do things we’re working on,” “Tries to understand how I see things,” “Really wants me to tell her just how I feel about things,” “Wants me to tell her about it if I don’t like the way she treats me,” “Allows me to tell her if I think my ideas are better than hers.” Ratings were made on the following 3-point scale (3-not at all true, 2-somewhat true, 1-very true; *M*s = 1.62–1.89, *SD*s = 0.45–0.47). Higher scores indicate more maternal overcontrol. The composite was internally reliable (αs = 0.70–0.84) and moderately stable between successive time points (*r*s = 0.26–0.52, *p*s *<* 0.01).

## 3. Results

### 3.1. Analytic Overview and Stability Path Results

**Figure 1:**
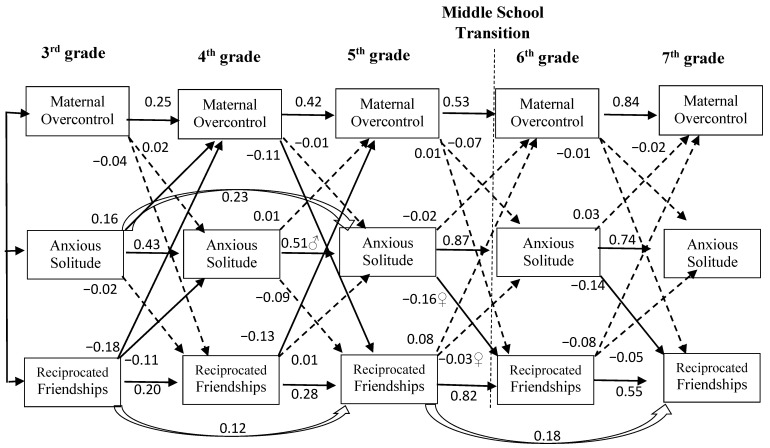
Autoregressive cross-lagged panel analysis of peer-reported anxious solitude and number of reciprocated friendships, and youth-reported maternal overcontrol from third through seventh grade. *n* = 230. χ^2^(60) = 89.69, *p* = 0.008; *NC* = 1.50; *CFI* = 0.96; *RMSEA* = 0.05, *p*-*close* = 0.60. Solid and outlined arrows indicated *significant* paths (*p* < 0.01 or *p* < 0.05). Dashed arrows indicated *non-significant* paths. Path coefficients are standardized. Gender moderation: ♂ = path stronger for boys than girls, ♀ = negative path for girls significantly differs from path for boys (gender-specific coefficients appear in the text). Assessments occurred in the fall of third grade and the spring of each subsequent grade.

To evaluate *Chronic Stress effects, Stress Generation effects*, and the overarching *Transactional Model* of anxious solitude development we constructed an *autoregressive cross-lagged panel analysis model* with AMOS 25 [37] (see Figure 1). This analytic model tested cross-lagged effects among the three focal variables (anxious solitude, reciprocated friendship, and maternal overcontrol) while accounting for the stability of each variable across the five time points from 3rd to 7th grade. This analytic model yielded path coefficients which estimated *incremental change* in each variable over time (increase or decrease; e.g., increase in the level of a variable from one assessment wave to the next which was predicted by a variable at an earlier wave). A *Chronic Stress Model* would be supported if the *autoregressive cross-lagged panel analysis model* reveals significant peer and/or maternal cross-lagged prediction of incremental increase in subsequent youth anxious solitude without support for the reverse direction of effect [9]. A *youth Stress Generation Model* would be supported if the autoregressive cross-lagged panel analysis model reveals youth anxious solitude as a significant cross-lagged predictor of subsequent incremental decrease in reciprocated friendship or subsequent incremental increase in maternal overcontrol over time without support for the reverse direction of effect [9]. A *Transactional Model* would be supported by significant paths consistent with both *Chronic Stress* and *Stress Generation Models*.

The autoregressive cross-lagged panel analysis model was constructed such that each variable was specified for each grade with all coefficients freely estimated. To test the proposed effects, we initially estimated the model with all stability and cross-lagged paths from *adjacent* time points. All adjacent stability paths were positive and significant as expected. Significant cross-lagged paths between focal variables in the final model are described below.

We next evaluated *non-adjacent* stability and cross-lagged paths because they would also be consistent with the effects of interest. The following significant non-adjacent paths were added to the model: stability paths from 3rd to 5th grade for both reciprocated friendship and anxious solitude, as well as the stability path from 5th to 7th grade for reciprocated friendship.

### 3.2. Model Fit

The final model (see Figure 1) fit the data well: χ^2^(60) = 89.69, *p* = 0.008; *NC* = 1.50; *CFI* = 0.96; *RMSEA* = 0.05, *p*-*close* = 0.60. Model fit was evaluated with chi-square (χ^2^), Normed Chi-square (*NC*), Comparative Fit Index (*CFI*), and Root Mean Square Residual Error of Approximation (*RMSEA*). Acceptable fit was judged according to criteria recommended by Hu and Bentler [38]. The chi-square is a test of “badness of fit,” with significant values indicating that the model fits the data poorly. However, because the chi-square statistic is sensitive to sample size, significant values are often the product of large sample size rather than poor model fit. The *NC* statistic corrects for this sensitivity by dividing the chi-square by the model’s degrees of freedom. *NC* values of 2 or less are considered acceptable [39]. The *CFI* is a discrepancy function adjusted for sample size. It ranges from 0 to 1 with a larger value indicating better model fit. *CFI* values of 0.90 or more indicate acceptable model fit. The *RMSEA* is another “badness of fit” index; but it corrects for model complexity. *RMSEA* values that are not significantly different from zero (defined as less than 0.06) are considered to be acceptable. According to all three latter criteria (*NC*, *CFI, RMSEA*) the model fit the data well.

### 3.3. Gender Moderation of Stability Paths

Each of the autoregressive cross-lagged panel analytic model’s (Figure 1) stability and cross-lagged paths was tested for gender differences. Gender differences were *not* supported for the majority of model paths. Nonetheless, significant gender differences emerged in one stability path and two cross-lagged paths involving anxious solitude and reciprocated friendship: boys compared to girls demonstrated more stability in anxious solitude from 4th to 5th grade (boy = 0.67 vs. girl = 0.37, χ^2^∆ (1) = 10.13, *p* < 0.01). Gender differences involving cross-lagged effects between anxious solitude and reciprocated friendship are described in the next section.

### 3.4. Cross-Lagged Paths between Reciprocated Friendship and Anxious Solitude with Gender Moderation

In support of *peer-driven Chronic Stress effects*, fewer reciprocated friendships in third grade predicted a significant incremental *increase* in youth anxious solitude in fourth grade (Figure 1; −0.11, *p* < 0.05). Additionally, having fewer friends in fifth grade predicted an incremental *increase* in anxious solitude after the middle school transition in sixth grade for *girls* but not for boys (girl = −0.15 vs. boy = 0.13, χ^2^∆ (1) = 5.81, *p* < 0.05).

In support of *youth Stress Generation effects*, the reverse direction of effects indicated that youth anxious solitude predicted incremental *decreases* in number of reciprocated friendships after the middle school transition from fifth to sixth grade (−0.16, *p* < 0.01), and from sixth to seventh grade (−0.14, *p* < 0.01). However, the first of these effects was qualified by gender, such that anxious solitude in fifth grade predicted fewer friends after the middle school transition in sixth grade for *girls* but *not* for *boys* (girl = −0.27 vs. boy = 0.00, χ^2^∆ (1) =4.30, *p* < 0.05). These combined *peer-driven Chronic Stress* and *youth Stress Generation effects* support an overall *Transactional Model* of anxious solitude development.

### 3.5. Cross-Lagged Paths between Anxious Solitude and Maternal Overcontrol

*Maternally-driven Chronic Stress effects* were *not* supported in mother-child relations (maternal overcontrol did *not* predict significant incremental change in youth anxious solitude over time). However, in support of *youth Stress Generation effects*, youth anxious solitude in third grade evoked an incremental increase in maternal overcontrol in fourth grade (0.16, *p* < 0.01) as expected. Although only *youth Stress Generation effects* were supported in the relations between anxious solitude and maternal overcontrol over time, these effects are also compatible with an overall *Transactional Model* of anxious solitude development involving relations with both friends and mothers.

### 3.6. Cross-Lagged Paths between Reciprocated Friendship and Maternal Overcontrol

Results also support systematic effects between youth’s relations with friends and mothers. In support of *peer-driven relational Stress Generation effects,* youth’s participation in few reciprocated friendships in third and fourth grades predicted incremental *increases* in their mother’s overcontrol in the subsequent grades (−0.18, *p* < 0.01 and −0.13, *p* < 0.05, respectively). Then this direction of effect reversed such that mothers’ overcontrol in fourth grade predicted an incremental *decrease* in their child’s number of reciprocated friendships in fifth grade (−0.11, *p* < 0.05; *maternally-driven relational Stress Generation effects*). These *relational Stress Generation effects* support an overall *Transactional Model* of anxious solitude development in which youth’s relations with friends impact their relations with their mothers, and subsequently youth’s relations with their mothers impact their friendships.

### 3.7. Mediation

Because there was the potential for mediation among all three variables, mediation was evaluated with bootstrapping in Mplus. Results suggest broad interrelations among the three variables over time, but do *not* support mediation paths at particular time points.

### 3.8. Effect Sizes

Lastly, in order to characterize effect size for each variable over time, we estimated the amount of variance accounted for with an *R*2 statistic for each variable in the autoregressive cross-lagged panel analytic model at each time point (see Table 3). The *R*2 at each time point indicates the total variance accounted for by all adjacent and non-adjacent stability and cross-lagged paths contributing to the variable at that point in time.

For anxious solitude, the model accounted for about a quarter to over half the variation over time. This relatively large effect is perhaps fitting as the main purpose of the model was to evaluate the contribution of key friend and maternal influences on the development of youth anxious solitude from middle childhood through early adolescence. In contrast, the model accounted for comparatively less but nonetheless significant medium-sized variation in reciprocated friendship (five percent to a little under half) and maternal overcontrol (seven percent to just over a quarter). This is perhaps not surprising because the model was intended to evaluate the contribution of youth anxious solitude to the development of these interpersonal relations over time, but not necessarily to provide an evaluation of key factors contributing to the development of these interpersonal relations over time.

### 3.9. Relations between Anxious Solitude and Reciprocated Friendship While Controlling for Peer Exclusion

In order to evaluate whether the effects of friendship made a unique contribution to the development of anxious solitude while accounting for the effects of peer exclusion, we computed a supplemental autoregressive cross-lagged panel analytic model in which we evaluated the effects of both reciprocated friendship and peer exclusion on the development of anxious solitude over time (power was insufficient to conduct a four-variable version of the existing autoregressive cross-lagged panel analytic model). The model fit the data well: χ^2^(61) = 102.78, *p* < 0.001; *NC* = 1.68; *CFI* = 0.97; *RMSEA* = 0.05, *p*-*close* = 0.32; *n* = 230.

In regard to *peer-driven Chronic Stress effects*, results indicated that, after accounting for the effects of peer exclusion, anxious solitude in 3rd grade no longer significantly predicted an incremental increase in anxious solitude in 4th grade. Nonetheless, having fewer friends in 5th grade still tended to predict an incremental *increase* in anxious solitude after the middle school transition in 6th grade for *girls* but *not* for *boys* (girl = −0.06 vs. boy = 0.08, χ^2^∆ (1) = 3.15, *p* < 0.10).

Also, in regard to *youth Stress Generation effects*, while accounting for peer exclusion, anxious solitude still predicted incremental decreases in youth’s number of reciprocated friends after the middle school transition from 5th to 6th grade, and 6th to 7th grade. Consistent with the original model, the first of these effects was qualified by gender, such that anxious solitude in 5th grade predicted fewer friends after the middle school transition in 6th grade for *girls* but *not boys* (girl = −0.28 vs. boy = 0.13, χ^2^∆ (1) = 7.94, *p* < 0.05).

## 4. Discussion

Results of this investigation support a *Transactional Model* of anxious solitude development including (1) *peer-driven Chronic Stress effects* in which having few friends contributes to increasing youth anxious solitude over time, (2) *youth Stress Generation effects* in which youth anxious solitude in turn contributes to a decreasing number of mutual friends in middle school and increasing maternal overcontrol during elementary school, as well as (3) *relational Stress Generation effects* in which mutually-exacerbating transactions occur between youth’s relationship partners (friends and mothers). These results make an important contribution to the literature because comprehensive evaluations of direction of effect between youth anxious solitude and peer and parent influences over time are rare. Our comprehensive analysis provides evidence for *peer-driven Chronic Stress effects*, *youth Stress Generation effects*, as well as *Relational Stress Generation effects* in an overarching *Transactional Model* of anxious solitude development. Additionally, modelling transactions between youth’s friends and mothers is particularly novel. Below we first discuss relations between youth anxious solitude and friendship, including gender differences and effects that remain significant after accounting for peer exclusion; next we discuss relations between anxious solitude and maternal overcontrol; and finally we discuss relations between youth’s friends and mothers.

### 4.1. Relations between Youth Anxious Solitude and Friendship

Having few mutual friends contributed to incremental increase in youth anxious solitude in mid elementary school and also—for girls—after the middle school transition (*peer-driven Chronic Stress effects*), but youth anxious solitude in turn contributed to decreasing numbers of mutual friends after the middle school transition, again especially for girls (*youth Stress Generation effects*). These findings suggest that youth’s unmet needs for close relationships with peers play a role in growth of their anxious solitude in the middle childhood to early adolescent period. This finding supports our expectation that losing mutual friends contributes to anxiety in approaching and interacting with peers over time. Likewise, anxious solitary tendencies appear to contribute the dissolution of some mutual friendships overtime, perhaps because friends find increasingly anxious youth to be less satisfying as friends and prefer better-adjusted friends.

#### 4.1.1. Gender Differences in Relations between Anxious Solitude and Friendship after the Middle School Transition

Why did stronger negative transactions between anxious solitude and mutual friendship occur after the transition to middle school for girls compared to boys? Anxious solitude contributed to losing mutual friends (and not replacing them) after the middle school transition in sixth grade for girls but not for boys (although the same effect from 6th to 7th grade was significant for both sexes). Likewise, losing friends in fifth grade contributed in growth in youth anxious solitude after the middle school transition in sixth grade for girls but not for boys. There may be several social dynamics which together contributed to this gendered pattern of findings.

First, girls spend more time interacting in friendship dyads, whereas boys spend more time interacting in groups [40]. Therefore, girls may feel more strongly invested in friendships, and friendships may have a stronger impact on their self-evaluations and social adjustment. Girls who have difficulty maintaining friendships may develop social insecurities and become increasingly socially anxious. Likewise, anxious solitary girls may experience particular difficulty maintaining friendships after the middle school transition as the friendships of anxious solitary youth are characterized by lower quality [19,20], existing and potential friends have more friendship partners to choose from, and there is an drive to find one’s place among restructured peer groups [30].

Second, the transition to middle school is known to be more stressful for girls than boys [41], in part because it corresponds more closely to the pubertal transition for many girls. Consequently, many young adolescent girls must cope with multiple transitions simultaneously. Anxious solitary girls, who already have a tendency towards social anxiety, may find the physical changes which accompany puberty and their social ramifications in the peer group (e.g., increased attention and teasing from boys and competition with other girls) to be particularly challenging.

Third, the relatively low rates of peer interaction that are typical of anxious solitary youth are likely to limit the normative development of their social skills, and this is likely to compound over time. Youth gain critical perspective-taking skills from social interaction, which form a basis for their social skills [42]. Social skills evolve rapidly in early adolescence. Consequently, growth in anxious solitary girls’ social skills may rely chiefly on interactions with friends, and they may increasingly fall behind peer norms for social skill if they lose friends in the middle school period.

#### 4.1.2. Relations between Anxious Solitude and Friendship after Accounting for Peer Exclusion

In this investigation we also evaluated the impact youth’s friendships (or the lack thereof) on the development of their anxious solitude while accounting for the effects of group-level peer exclusion [2] because exclusion contributes to growth in anxious solitude over time [9,15]. Results indicated that mutual friendships in third grade no longer significantly predicted incremental increase in anxious solitude in fourth grade after accounting for the effects of peer exclusion (*peer-driven Chronic Stress effects* of friendship were *not* unique), but that anxious solitude still predicted incremental decreases in mutual friendship after the middle school transition after accounting for peer exclusion (*youth Stress Generation effects* were unique). It is perhaps not surprising that having few mutual friendships no longer predicted incremental increase in anxious solitude after accounting for peer exclusion because peer exclusion has repeatedly been found to exert robust effects on anxious solitude over time [9].

Nonetheless, the unique contribution of anxious solitude to incremental losses in mutual friendship after the middle school transition even after accounting for peer exclusion is an important finding. This suggests that anxious solitude may incur increasing costs to youth’s friendships as they mature. This may occur because increasingly mature social and perspective taking skills are expected by friends in order to be successful in maintaining (or replacing) these relationships. Interactions with friends are a critical training ground for the growth of these skills over time. As anxious solitary youth miss out on interactions with friends, they may fall behind as their middle school peers rapidly become more sophisticated in their interactions with friends, and in the knowledge of peer culture and norms that is derived from such interaction.

### 4.2. Relations between Youth Anxious Solitude and Maternal Overcontrol

Present results suggest that youth anxious solitude in third grade evoked increased maternal overcontrol in fourth grade (*youth Stress Generation effect*), but the reverse direction of effect was not supported (*no maternally-driven Chronic Stress effect*). The present findings expand evidence that youth’s social withdrawal can evoke parenting (barring confounding factors). Youth anxious solitude likely arouses maternal concern, which may be expressed through overcontrol. Mothers may find their child’s anxious solitary behavior taxing and attempt to manage it by discouraging their child from expressing anxious concerns and eliminating their child’s exposure to potentially anxiety-provoking situations. For example, in an attempt to prevent her child from becoming anxious, a mother may not allow her child to spend the night as someone else’s house or attend sleep-away camp with classmates. Although these restrictions are well-intentioned responses to the youth’s anxiety and aimed at preventing the youth from becoming anxious, they are likely to have the unintended effect of limiting the youth’s exposure to social interaction, thus preventing the youth from having fear-disconfirming social experiences and limiting opportunities for interactions that could help build and maintain friendships.

Although the effects of parenting on youth’s social withdrawal and anxiety has received substantial research attention [23,24,43,44], in the present study maternal overcontrol did *not* significantly contribute to incremental growth in youth anxious solitude in the middle childhood to early adolescent period. However, this does not rule out the possibility that maternal overcontrol may have contributed to the development of youth’s anxious solitude earlier in development.

### 4.3. Transactions between Youth’s Friends and Mothers

Similarly, evidence suggests that youth’s involvement in few friendships also evoked mothers’ overcontrol in middle childhood. This suggests that mothers may interpret friendships as a barometer for their child’s psychosocial wellbeing and become concerned when their child loses friendships over time. Maternal overcontrol, in turn, subsequently exacerbated youth’s loss of friendships in elementary school.

### 4.4. Conclusions and Limitations

Taken together, in support of an overarching *Transactional Model*, evidence suggests that having few mutual friends contributed to increased anxious social behavior in youth and overcontrolling parenting in their mothers, and subsequently these qualities of youth and their mothers further diminished youth’s mutual friendships over time. Unfortunately, these results suggest a vicious cycle in which the responses of both youth and their mothers (increase in youth anxious solitude and maternal overcontrol) exacerbate the situation by contributing to further losses of youth’s mutual friendships over time.

Although these results enhance our understanding of the interplay between youth’s anxious solitude and their relationships with their friends and mothers from middle childhood through early adolescence, it is nonetheless important to acknowledge the limitations of the current investigation. Although our analytic approach was well-suited to testing *Chronic Stress*, *Stress Generation*, and *Transactional Models* of anxious solitude development, as a variable-centered approach it likely does not describe patterns of development that deviate from the predominant patterns demonstrated in the sample. Person-centered analyses are better suited to describing patterns of development that deviate from predominant patterns (see relevant examples [15,16]).

Additionally, our sample was drawn from a suburban to rural region of the southeastern United States in which some American ethnic subgroups that were representative of the area were reasonably well-represented (European American, Latinx, and African American youth), but others less prevalent in the area were not well-represented (Asian American and Native American Youth). Thus, caution should be exercised in generalizing the present finings to populations not represented in the sample.

Future research should continue to model potential evocative effects of youth’s anxious solitude and peer relations on parenting in the context of transactional models. Likewise, it will be important in future research to consider the effects of fathers as well as mothers on the development of anxious solitude.

### 4.5. Clinical Implications

The overall pattern of results suggests that intervention with anxious solitary youth, and particularly girls, prior to and during the middle school transition, could prevent loss of mutual friendships after the middle school transition such as those observed in this investigation. This may be an important way to support anxious solitary girls’ adjustment to middle school and overall psychosocial health during the early adolescent period. Nonetheless, such interventions should also include a focus on preventing peer exclusion [45] given its robust relations with anxious solitude over the middle childhood to early adolescent period.

## Figures and Tables

**Table 1:** Definition of effects and model.

Effect or Model	Definition
*Transactional Model*	*Interplay between multiple youth and social influences over time*
*Chronic Stress effects*	*Stress with social partners contributes to youth AS*
Peer-driven	Stress with friends contributes to youth AS
Maternally-driven	Stress with mother contributes to youth AS
*Stress Generation effects*	*Youth or social partner contributes to stress with social partners*
Youth	Youth AS contributes to stress with social partners
Relational	Transactions occur between youth’s social partners

**Table 2:** Means, standard deviations, sample size, and Pearson intercorrelations for all study variables from third through seventh grade.

	Gender	Anxious Solitude (AS) Peer Report					Reciprocated Friends (RF) Peer Report					Maternal Overcontrol (MO) Youth Report				
		3rd Gr	4th Gr	5th Gr	6th Gr	7th Gr	3rd Gr	4th Gr	5th Gr	6th Gr	7th Gr	3rd Gr	4th Gr	5th Gr	6th Gr	7th Gr
***M*** (% girls)	57%	0.45	0.38	0.29	0.21	0.30	1.45	1.56	2.8	2.68	2.95	1.89	1.81	1.62	1.73	1.71
***SD***	−	1.03	1.11	1.16	1.21	1.50	1.26	1.24	1.93	2.14	2.38	0.46	0.45	0.47	0.46	0.45
***n***	230	230	197	226	193	176	230	197	222	188	176	161	182	208	180	171
AS 3rd Gr	−0.08	1.00														
AS 4th Gr	0.01	0.46 **	1.00													
AS 5th Gr	0.10	0.45 **	0.61 **	1.00												
AS 6th Gr	0.14 †	0.40 **	0.50 **	0.59 **	1.00											
AS 7th Gr	0.06	0.39 **	0.40 **	0.52 **	0.77 **	1.00										
RF 3rd Gr	−0.18 **	−0.24 **	−0.24 **	−0.18 **	−0.18 *	−0.16 *	1.00									
RF 4th Gr	−0.17 *	−0.06	−0.21 **	−0.10	−0.14	−0.07	0.22 **	1.00								
RF 5th Gr	−0.16 *	−0.09	−0.18 *	−0.24 **	−0.15 *	−0.21 **	0.26 **	0.33 **	1.00							
RF 6th Gr	−0.25 **	−0.17 *	−0.19 *	−0.26 **	−0.25 **	−0.24 **	0.20 **	0.30 **	0.31 **	1.00						
RF 7th Gr	−0.24 **	−0.16 *	−0.28 **	−0.28 **	−0.31 **	−0.27 **	0.17 *	0.32 **	0.37 **	0.61 **	1.00					
MO 3rd Gr	0.02	0.02	0.00	0.05	0.03	0.06	−0.07	−0.02	−0.13	−0.01	−0.07	1.00				
MO 4th Gr	0.08	0.21 **	0.12	0.06	0.08	0.02	−0.23 **	−0.03	−0.15	−0.15	−0.13	0.26 **	1.00			
MO 5th Gr	0.08	0.05	0.07	0.11	0.00	0.02	−0.15 *	−0.14	−0.17 *	−0.11	−0.18 *	0.22 **	0.42 **	1.00		
MO 6th Gr	0.11	0.08	0.13	0.03	0.04	0.01	−0.03	0.02	0.03	−0.07	−0.06	0.22 *	0.51 **	0.52 **	1.00	
MO 7th Gr	0.09	0.10	0.13	0.03	0.03	0.04	−0.15	−0.08	−0.07	−0.11	−0.13	0.22 *	0.36 **	0.44 **	0.47 **	1.00

*n* for *r*s = 118–230. Gender: girl −1, boy 1. Gr = grade in school. 2-tailed significance levels: † < 0.10, * *p* < 0.05. ** *p* < 0.01. Assessments were conducted in the Fall of third grade and each subsequent Spring. The middle school transition occurred in the fall of sixth grade. χ^2^(601) = 633.04, *ns*. Consequently, all available data were included in our main analyses with Full Information Maximum Likelihood (FIML) estimation.

**Table 3:** Effect size: Variance accounted for (*R*2) by variable and grade in school.

Variable	*R2* by Grade in School			
	4th	5th	6th	7th
AS	0.22	0.42	0.28	0.56
RF	0.05	0.15	0.12	0.45
MO	0.15	0.20	0.28	0.07

AS = Anxious Solitude, RF = Reciprocated Friendship, MO = Maternal Overcontrol.

## Data Availability

Queries related to the data reported here should be directed to Heidi Gazelle.
